# Ultrasound-driven piezoelectric current activates spinal cord neurocircuits and restores locomotion in rats with spinal cord injury

**DOI:** 10.1186/s42234-020-00048-2

**Published:** 2020-06-01

**Authors:** Shuai Li, Monzurul Alam, Rakib Uddin Ahmed, Hui Zhong, Xiao-Yun Wang, Serena Ng, Yong-Ping Zheng

**Affiliations:** 1 Department of Biomedical Engineering, The Hong Kong Polytechnic University, Hung Hom, Kowloon, Hong Kong, China; 2 Department of Integrative Biology and Physiology, University of California, Los Angeles, USA; 3 Guangdong Work Injury Rehabilitation Center, Guangzhou, China; 4 Community Rehabilitation Service Support Centre, Hospital Authority, Hong Kong, China

**Keywords:** Neurostimulation, Neuromodulation, piezoelectric, Ultrasound, Epidural, Spinal cord injury

## Abstract

**Background:**

Neuromodulation via electrical stimulation (ES) is a common technique to treat numerous brain and spinal cord related neurological conditions. In the present study, we examined the efficacy of piezoelectric stimulation (pES) by a custom miniature piezostimulator to activate the spinal cord neurocircuit in comparison with conventional epidural ES in rats.

**Methods:**

Stimulation electrodes were implanted on L2 and S1 spinal cord and were connected to a head-plug for ES, and a piezostimulator for pES. EMG electrodes were implanted into hindlimb muscles. To generate piezoelectric current, an ultrasound beam was delivered by an external ultrasound probe. Motor evoked potentials (MEPs) were recorded during the piezoelectric stimulation and compared with the signals generated by the ES.

**Results:**

Our results suggest that ultrasound intensity as low as 0.1 mW/cm^2^ could induce MEPs in the hindlimbs. No significant difference was found either in MEPs or in muscle recruitments for ES and pES. Similar to ES, pES induced by 22.5 mW/cm^2^ ultrasound restored locomotion in paralyzed rats with complete thoracic cord injury. Locomotion EMG signals indicated that pES works same as ES.

**Conclusion:**

We propose piezoelectric stimulation as a new avenue of neuromodulation with features overtaking conventional electrical stimulation to serve future bioelectronic medicine.

Video abstract.

## Background

According to the latest report, worldwide, every 1 out of 6 people is currently living with a neurological condition (Feigin et al. 2020). This number is even growing. For instance, about 0.5 million people are becoming paralyzed each year due to spinal cord injury (SCI) (World Health Organization 2013). These patients are not only suffering from limb paralysis, depending on the location and the severity of the injury, they also suffer with bladder, bowel, respiratory and cardiovascular dysfunctions (Harvey 2016; Sluka and Walsh 2003). Although there is no cure for the paralysis resulting from SCI yet, fortunately, some neuromuscular stimulation such as functional electrical stimulation (FES) and epidural electrical stimulation (ES) to the spinal cord have positive impacts on functional restorations (Ridler 2018; Shah et al. 2016).

Spinal cord contains complex neurocircuits that are capable of processing information on their own with no or minimal inputs from the brain (Lyon et al. 2005). For instance, spinal reflexes are merely involuntary and nearly instantaneous movement responses in response to particular sensory stimulus. Spinal locomotor circuit, also known as central pattern generator (CPG) which can produce cyclic synergies to the periphery (Li et al. 2013). ES to the spinal cord is a very effective way to activate such circuits which restores locomotion and even voluntary control over the paralyzed leg muscles in SCI patients (Willyard 2019).

Most neurostimulation utilizes square pulse-shaped electric voltage or current delivered from an electrical stimulator powered by a battery or external power source (Merrill et al. 2005). However, some non-rectangular waveforms were found to work more efficiently (Sahin and Tie 2007). No matter what kind of shape is applied, it generally requires a neurostimulator to be implanted or connected to the patient (Ridler 2018). Most implantable stimulators require batteries as their power source which significantly increases the size (about 80%) of the implant (Amar et al. 2015). Furthermore, battery-powered stimulators require a secondary surgery as battery life is projected to be 5–10 years, depending on its discharge and recharge cycles (Kane et al. 2011; Loeb et al. 2006). In contrast, our recently developed piezoelectric stimulator (Alam et al. 2019) does not require any implanted power source; rather the power is delivered via ultrasound beam from an external ultrasound probe. This allows us to design a distributed stimulation system that can deliver piezoelectric currents to the targeted organ. In the present study, we investigate whether the piezoelectric current from a single piezostimulator can activate spinal cord neural circuit similar to conventional electric current and can similarly restore locomotion after paralysis.

## Methods

### Animals

Seven Spraque Dawley rats (245–262 g body-weight) were utilized in this study. All the surgical and experimental procedures were performed in accordance with the guidelines and approval of the Animal Subjects Ethics Sub-committee of the Hong Kong Polytechnic University.

### Piezoelectric stimulation system

Detailed developmental procedure of a piezoelectric stimulator utilized in this study is described elsewhere (Alam et al. 2019). In brief, a piezoelectric ceramic Barium Titanate (BaTiO_3_) with around 1 MHz resonance frequency and a signal conditioning circuit (Villard voltage doubler) were used to convert ultrasound signals into piezoelectric stimulation pulses. We utilized BaTiO_3_ in our design for it’s piezoelectric properties and non-toxic nature. All piezo-ceramics were first examined for their center frequency for generating maximum piezoelectric voltage (see Supplementary document). After prototyping, the entire stimulator was encapsulated with a biocompatible silicone coating as described previously (Alam et al. 2019). A pair of Teflon-coated stimulation wires (AS-632, Cooner Wire, United States) were connected to the piezostimulator for the delivery of stimulation current.

**Fig. 1:**
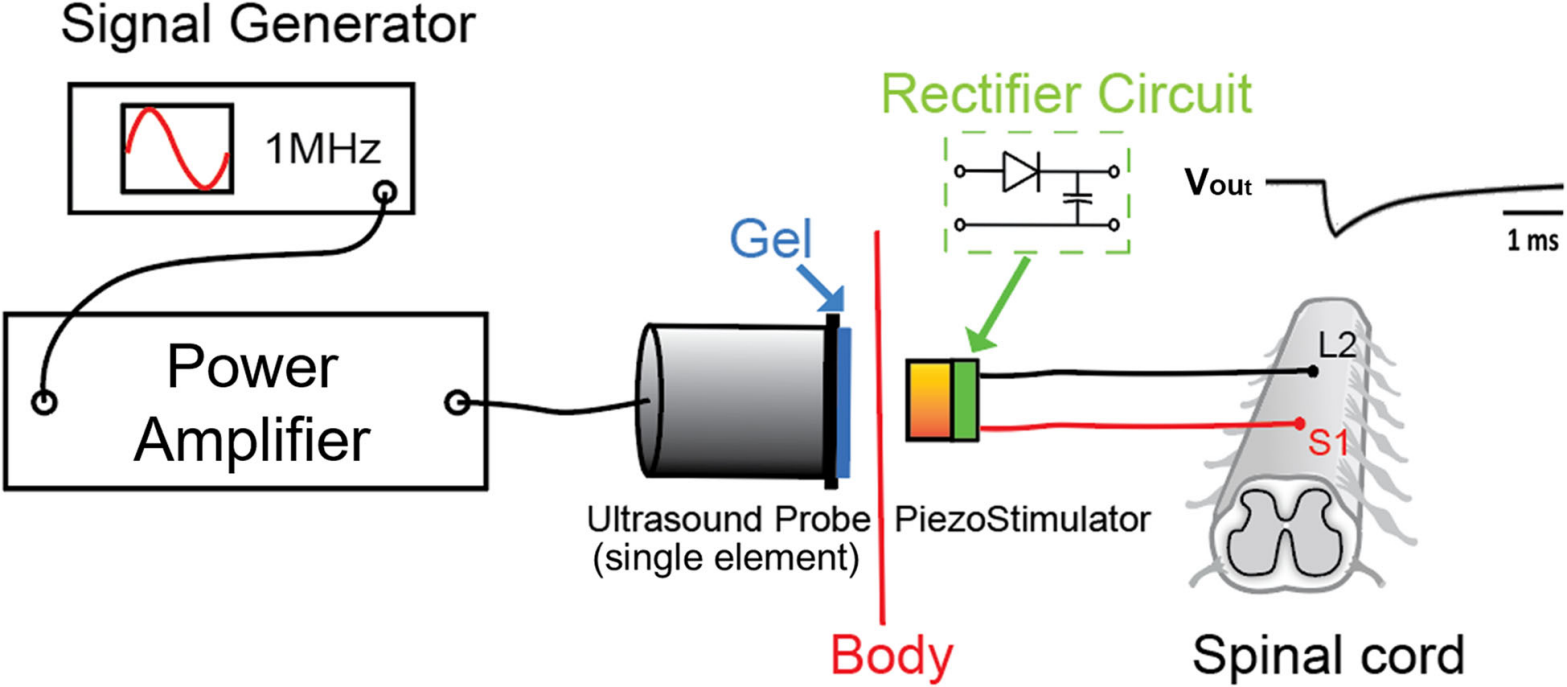
Schematic diagram of the piezoelectric stimulation (pES) experiment setup. The setup includes an external ultrasound energy transmitting system and implanted receiving and piezoelectric current generation module. A signal generator produces 200 μs bursts (1 MHz carrier frequency) of sinusoidal signal and feeds it into a power amplifier to drive an ultrasound probe to produce acoustic energy. With the aid of ultrasound gels, the acoustic energy was then transferred through the skin and was received by the piezostimulator implant. The implant contained a rectifier and filter circuit to convert the sinusoidal piezoelectric signal into a monophasic stimulation pulse for pES. This pES pulse was then delivered to the lumbosacral spinal cord (L2 and S1 levels) via Teflon-coated stimulation wires to activate the neural circuits

To power the piezostimulator implant, sinusoidal signal from a signal generator (AFG3021, Tekronix, United States) was fed to a 50-watt power amplifier (Dahan Radio Studio, China) to drive an ultrasonic probe (1 MHz, DOBO, China) that generated the external ultrasound signal (see Fig. 1). The amplitude and burst period of the ultrasound signal could be directly controlled by the signal generator. From this ultrasound source, the maximum output power of our piezoelectric stimulator was found to be 3.5 mW for an input power (electrical) of 1600 mW, suggesting the efficiency of about 0.22%. For epidural electrical stimulation (ES), we utilized a conventional isolated voltage stimulator (DS2A, Digitimer Limited, United Kingdom) and a constant current stimulator (DS3, Digitimer Limited, United Kingdom).

### Surgical procedure

Under aseptic conditions, the rats were anaesthetized with isoflurane gas (5%) which was maintained (1.5–2%) via a facemask throughout the surgery. The body temperature was maintained at 37 °C by using a homeothermic system (ThermoStar Homeothermic Monitoring System, RWD Life Science Co., Ltd., China). To implant the headplug on the skull for accessing the electrodes, a small incision was made and the muscles and fascia were removed laterally. After the skull was thoroughly dried, one 14-pin (7 channels) premade headplug with Teflon-coated stainless steel wires (AS632, Cooner Wire, United States) were securely attached to the skull with screws and dental cement. In the lower lumbar region, a longitudinal skin incision (2–3 cm) was made to place the wires subcutaneously. Another skin incision was made in the hindlimb bilaterally to pass the EMG wires to the soleus (Sol) and tibialis anterior (TA) muscles. A 27-gauze needle was used to pass the electrodes into each muscle belly. After placing the electrodes in the muscles, a small portion (~ 1 mm) of the Teflon coating was removed to make an EMG electrode. The electrodes were than anchored by using 4.0 Ethilon sutures. To relieve stress the EMG wires were coiled subcutaneously at the implantation site.

For a complete spinal transection, a mid-dorsal skin incision was made between the T6 and T10 spinal level. The paravertebral muscles overlying the vertebral column were retracted and a partial laminectomy was performed at the mid thoracic (~T7) level. The dura mater was removed from the midline by using a 29-gauze needle and sharp microscissors were used to transect the spinal cord completely. To coagulate the blood, gel foam was inserted into the gap. Two partial laminectomies were then performed to expose the spinal cord segments L2 and S1 to implant the epidural stimulation electrodes. The Teflon-coated stimulation wires were passed above the dura mater to the exposed regions of the spinal cord. Small portions (~ 1 mm) of the Teflon coating were removed to make stimulation electrodes, and were secured on the midline of the spinal cord at the L2 and S1 levels (see Fig. 1).

Analgesic Buprenorphine HCL (Buprenex®, 0.5 mg/kg, s.c.) and antibiotic Enrofloxacin (Baytril®, 0.5 mg/kg, s.c.) were administered before completion of the surgery and continued for a minimum of 3 days. The urinary bladders of all injured rats were expressed manually before being put into a temperature- and humidity-controlled incubator (ICU-1801, AEOLUS International Pet Products, United States). After recovering from the surgery, the rats were housed individually and their bladders were expressed manually three times per day. For faster recovery, fresh fruit and juice were provided in the cage.

### Locomotion training

First, the rats were trained to walk bipedally on a moving treadmill belt as described previously (Gerasimenko et al. 2006). Our custom locomotion training system includes a motor-driven treadmill and a body-weight support harness. Rats were trained 5 days/week for 4 weeks on bipedal stepping. Each training day (in the morning) consisted of 3 training sessions, and each session took around 15–20 min. Varying walking speeds (10, 12.5, 15 cm/s) were used to train the rats to find the best hindlimb swing phase.

### Evoked potentials recording

To record the evoked potentials (MEPs) in the hindlimb muscles response to ES and pES, ES was delivered by an external voltage stimulator (DS2A, Digitimer, United Kingdom), and pES was delivered by our custom implanted piezostimulator (Alam et al. 2019). Under general anaesthesia, stimulation was delivered at 0.2 Hz to induce MEPs on Sol and TA muscles of the both hinglimbs. The stimulation was increased until strong movement was visible. The incremental steps of the intensities were kept constant at 0.1 V for ES and 0.003 mW/cm^2^ for pES. The MEP signal was amplified (100×) and filtered (10 Hz-10 kHz, bandpass) by an analogue amplifier (Model 1700 Differential AC Amplifier, AM Systems, United States). The signal was then digitized at 5 ks/s by a data acquisition system (Power1401-3A, Cambridge Electronics Design Ltd., United Kingdom). The digitized data were visualized and recorded on a computer for further analysis via a software interface (Signal, Cambridge Electronics Design Ltd., United Kingdom).

### Bipedal locomotion from epidural spinal cord stimulation

To restore bipedal locomotion, epidural spinal cord stimulation was delivered at 40 Hz for both ES and pES. Since 200 μs epidural electrical spinal cord stimulation was found to be most effective for restoring bipedal locomotion in paralyzed rats (Ichiyama et al. 2005), the stimulating period was set to this value for both ES and pES (200 cycles). The stimulation intensity of ES was set for a clear hindlimb swing movements. The ES value was recorded and used to set the ultrasound intensity for pES. EMG and video were recorded during the stimulation-induced bipedal locomotion for further analysis.

### Data analysis and statistics

All the data were processed by custom computer programs developed in MATLAB (MathWorks Inc., United States). Peak-to-peak voltage (V_pp_) and area-under-the-curve (AUC) of a MEP signal were calculated and normalized. The acoustic intensity of the ultrasound signal was also calculated from the data from a hydrophone setup (see Supplementary document). Statistical significance (*P* < 0.05) was measured using Graphpad Prism (GraphPad Software Inc., United States). One-way ANOVA was used to compare the maximum piezoelectric voltage harvested from the piezostimulator at the different weeks after implantation. Unpaired t-test was used to detect if there was any significant difference between ES and pES threshold voltages. All the group data are reported as mean (± standard error).

## Results and discussion

**Fig. 2:**
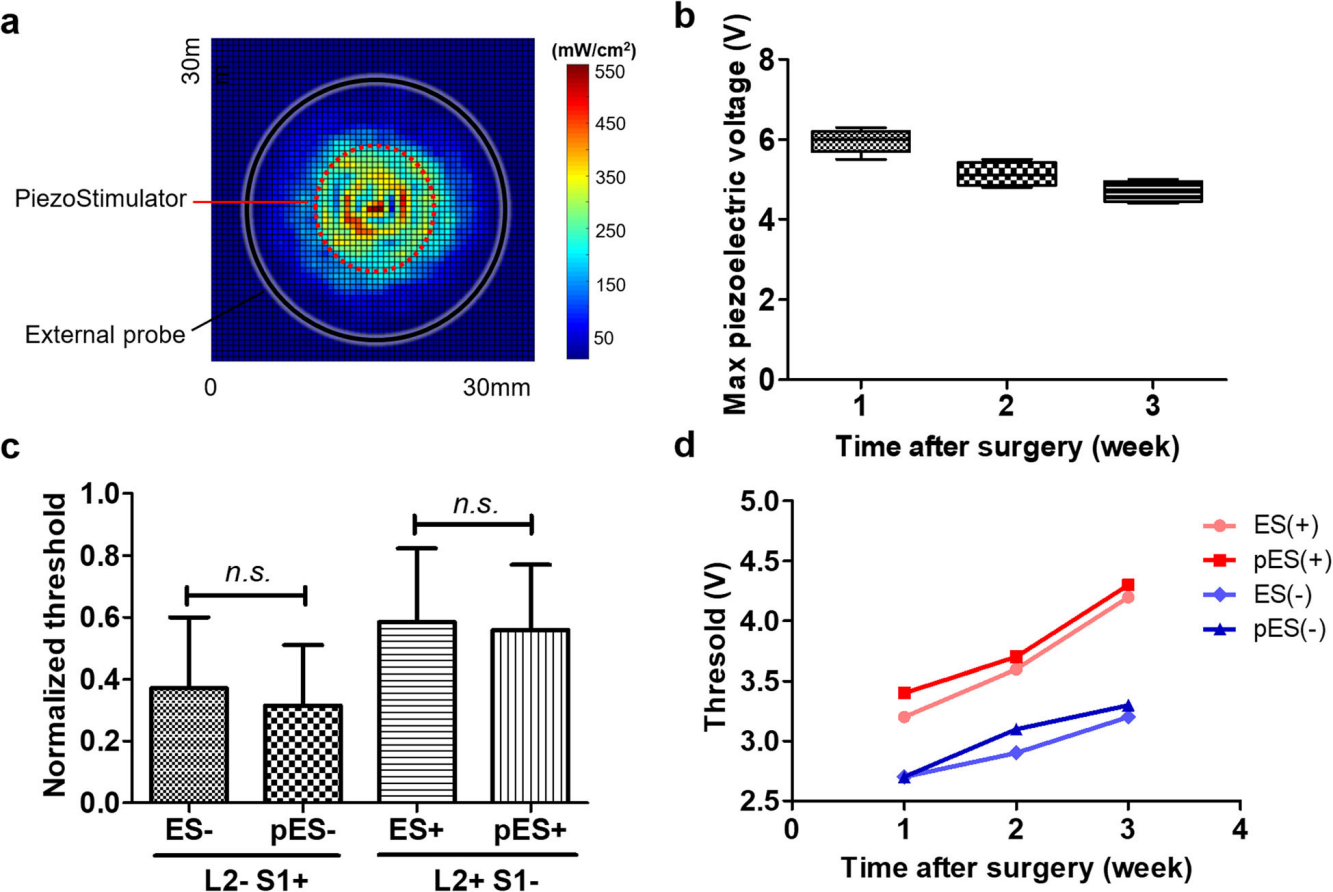
**a** Under PRF of 1 kHz, acoustic intensity-distributing map of the external ultrasound probe measured at a distance of 4 mm from its surface. The black circle indicates the size of ultrasound probe, and the red dotted circle indicates the size of the implanted piezoelectric stimulator. Acoustic intensities I_SPTA_ (spatial-peak temporal-average) was 561.9 mW/cm^2^ and I_SPPA_ (spatial-peak pulse-average) was 3.9 W/cm^2^ (Mechanical Index, MI was 0.26). **b** Maximum harvested piezoelectric pulse voltage from all healthy rats (*n* = 5). Maximum pES voltage was found to be stable for the first 3 weeks after implantation. Scar tissue formation and change in acoustic impedance might be the cause of the decreased piezoelectric voltage. **c** Comparisons between conventional electrical stimulation (ES) and pES threshold voltages to activate L2 and S1 spinal cord segments (both anodic: current from L2 to S1, and cathodic: current from S1 to L2). No significant difference was found in the threshold voltages between ES and pES for either anodic or cathodic stimulations. **d** Trends of ES and pES voltage thresholds over 3 weeks post-implantation in one intact rat. Both stimulation thresholds tend to increase over time, but clearly followed each other (ES vs. pES)

In the anaesthetized rats, stimulation threshold for generating an evoked movement was found to be from 3.6 to 4.0 V, or from 0.35 to 0.39 mA for ES, suggesting the electrode-tissue impedance of around 10.29 kΩ. As both ES and pES utilized the same epidural stimulation electrodes, the approximate power for the stimulation threshold could be calculated (1.23 ± 0.37 mW for ES, and 1.24 ± 0.39 mW for pES). Figure 2a shows the ultrasound intensity map of the external ultrasound probe measured in a custom ultrasound scanning system (see Supplementary document). For powering the piezostimulator implant, the ultrasound intensity of the ultrasound transducer was found to be 0.1 and 22.5 mW/cm^2^ (I_SPTA_) at 1 Mz for 200 cycles with PRF of 0.2 Hz and 40 Hz, respectively; and 3.9 W/cm^2^ (I_SPPA_) measured at 4 mm distance from the probe in water (MI = 0.26). All the ultrasound intensities were found much lower than the FDA safety limits (I_SPTA_ < 720 mW/cm^2^, either I_SPPA_ < 190 W/cm^2^ or MI < 1.9, Food and Drug Administration 2019). In these ultrasound intensities, the harvested maximum piezoelectric voltages were all larger than 4 V for all the weeks tested (Fig. 2b), and were found sufficient for spinal cord stimulation. There was, however, a slight drop of this harvested voltage over time, probably due to the increase of acoustic impedance inside the body due to tissue growth and scar formation after implantation. Tissue with high impedance can absorb more acoustic energy and may result barrier for ultrasound signals to reach deep inside the body (Speed 2001).

Hindleg movements were elicited in the anaesthetized rats when the spinal cord stimulation voltage crossed the motor threshold. The threshold voltage for conventional ES and the novel pES were measured for both anodic stimulation (+) where current passed from L2 to S1, and cathodic stimulation (−) where current passed from S1 to L2 spinal cord to examine if there was any difference of the stimulation thresholds. The normalized threshold values for all the rats at 1 week post-implantation are shown in Fig. 2c. No significant difference was found for either groups ES−/pES- (*p* = 0.906*,* t-test) or ES+/pES+ (*p* = 0.943, t-test), suggesting that the stimulations induced by ES and pES were equivalent. Furthermore, similar to our previous report (Shah et al. 2016), the threshold voltage for the anodic stimulation (+) appeared to be higher than that of the cathodic stimulation (−). The trends for the threshold voltages, however, increased over time as shown in Fig. 2d. But, encouragingly, both ES and pES thresholds demonstrated similar increasing trends suggesting their similarities. The threshold rising trends were probably due to the increase of epidural electrode impendence resulting from tissue and scar growth (Wilk et al. 2016).

**Fig. 3:**
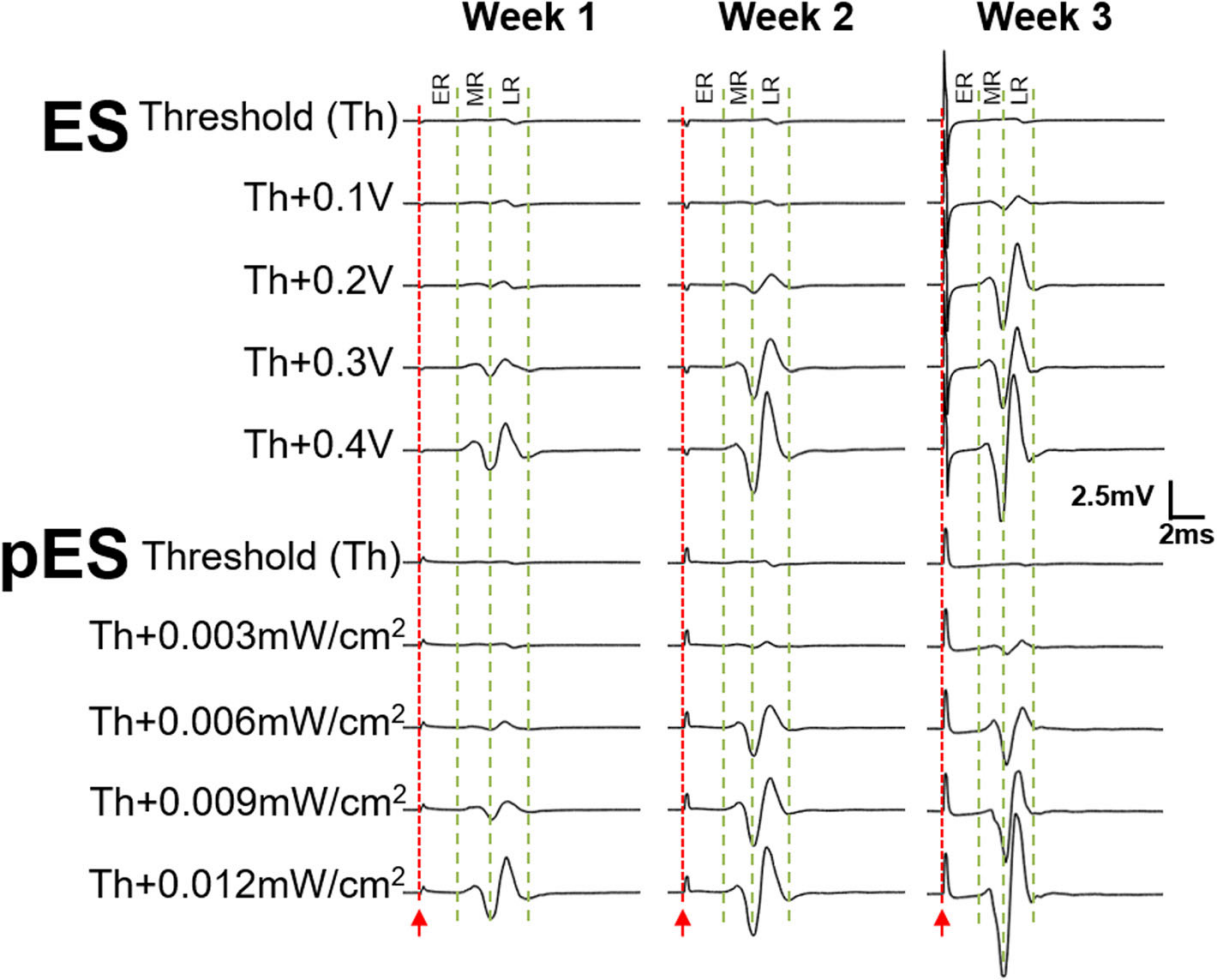
Motor evoked potentials (MEPs) of the tibialis anterior (TA) muscle activated by ES and pES stimulation pulses in the first 3 weeks after surgery. For different weeks, the stimulation threshold was different for both ES and pES. In each stimulation type (ES and pES), a constant input intensity (voltage or acoustic strength) was used to find if there was any difference of their MEPs. For ES, 0.1 V was the constant step of intensity, and I_SPTA_ of 0.003 mW/cm^2^ was the constant step of intensity for pES. Stimulation onset is indicated by a red line. Early (ER), middle (MR) and late (LR) responses are also marked with dotted lines

MEPs induced by conventional epidural ES generally consist of multiple components, such as an early (ER), middle (MR) and late (LR) responses related to direct motor response, and monosynaptic and polysynaptic reflex pathways as described previously (Lavrov et al. 2008). In the current study, both ES and pES induced MEPs in TA muscle at different weeks post-implantation as shown in Fig. 3. All MEPs were elicited with different stimulation voltages (for ES) or acoustic intensities (for pES) at or above the threshold (Th). At the threshold intensity, low or no MEP was observed, but increased at increasing stimulation intensities. The MEPs appeared consistent for both ES and pES. For both the stimulations (ES and pES), ER started at approximately 2–3 ms after the stimulation onset, and the latency for the MR ranged from approximately 5–6 ms, similar to a previous report (Gerasimenko et al. 2006). However, no LR responses were observed among these rats. This might be due to the anaesthetized condition of our rats (Lyon et al. 2005). With time, increased MEP signals were observed for both ES and pES for the same input voltage (ES) or intensity (pES), which could indicate a higher sensitivity of the spinal cord under the neurostimulation. Nonetheless, the MEPs elicited by both ES and pES were similar, both showing an increasing trend over time.

**Fig. 4:**
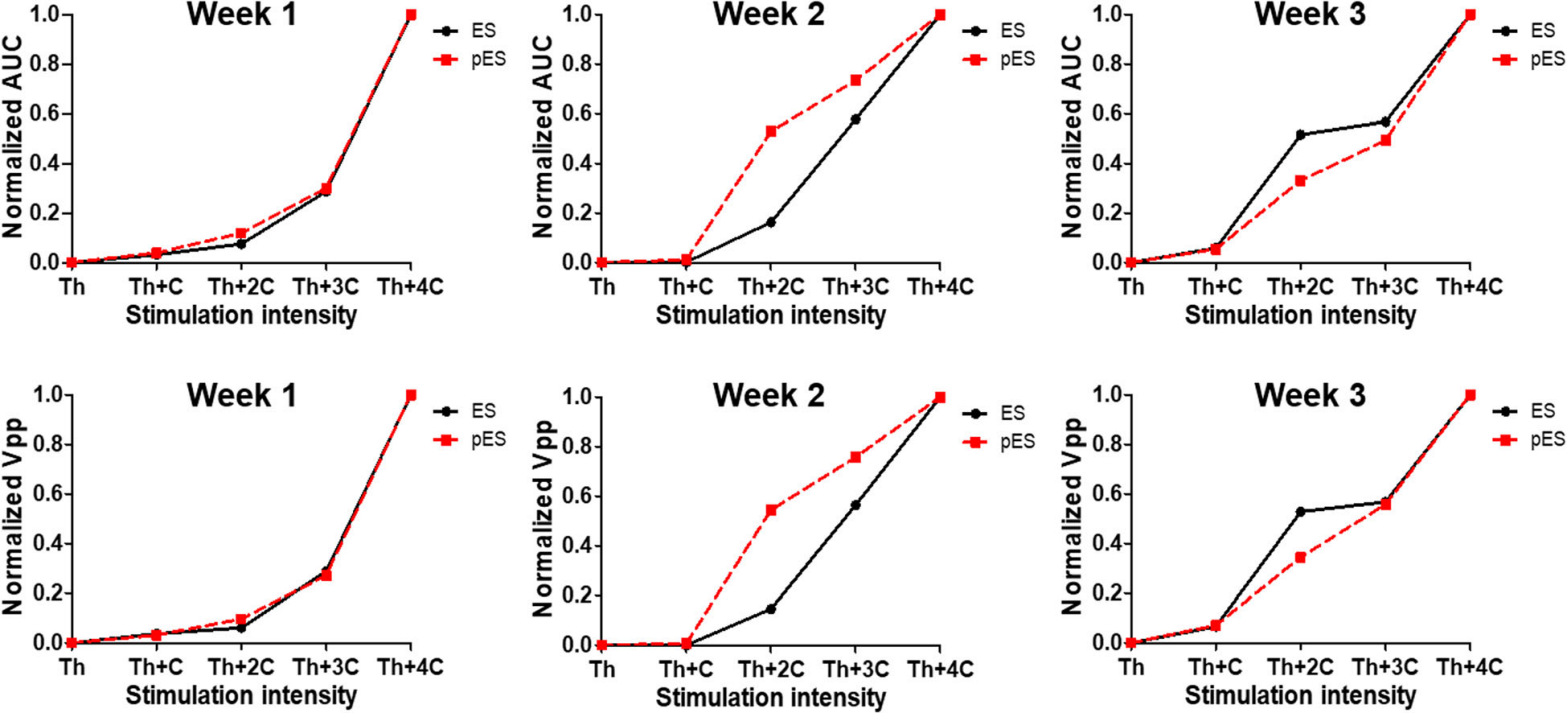
Normalized area-under-the-curve (AUC) and peak-to-peak voltage (V_pp_) were measured to enable a clear comparison between ES and pES MEPs. Week 1 and week 3 AUC and V_pp_ show similar recruitment curves of both ES and pES, while week 2 spinal cord seems to be more sensitive to the pES. C equals to 0.1 V for ES or 0.003 mW/cm^2^ for pES

**Fig. 5:**
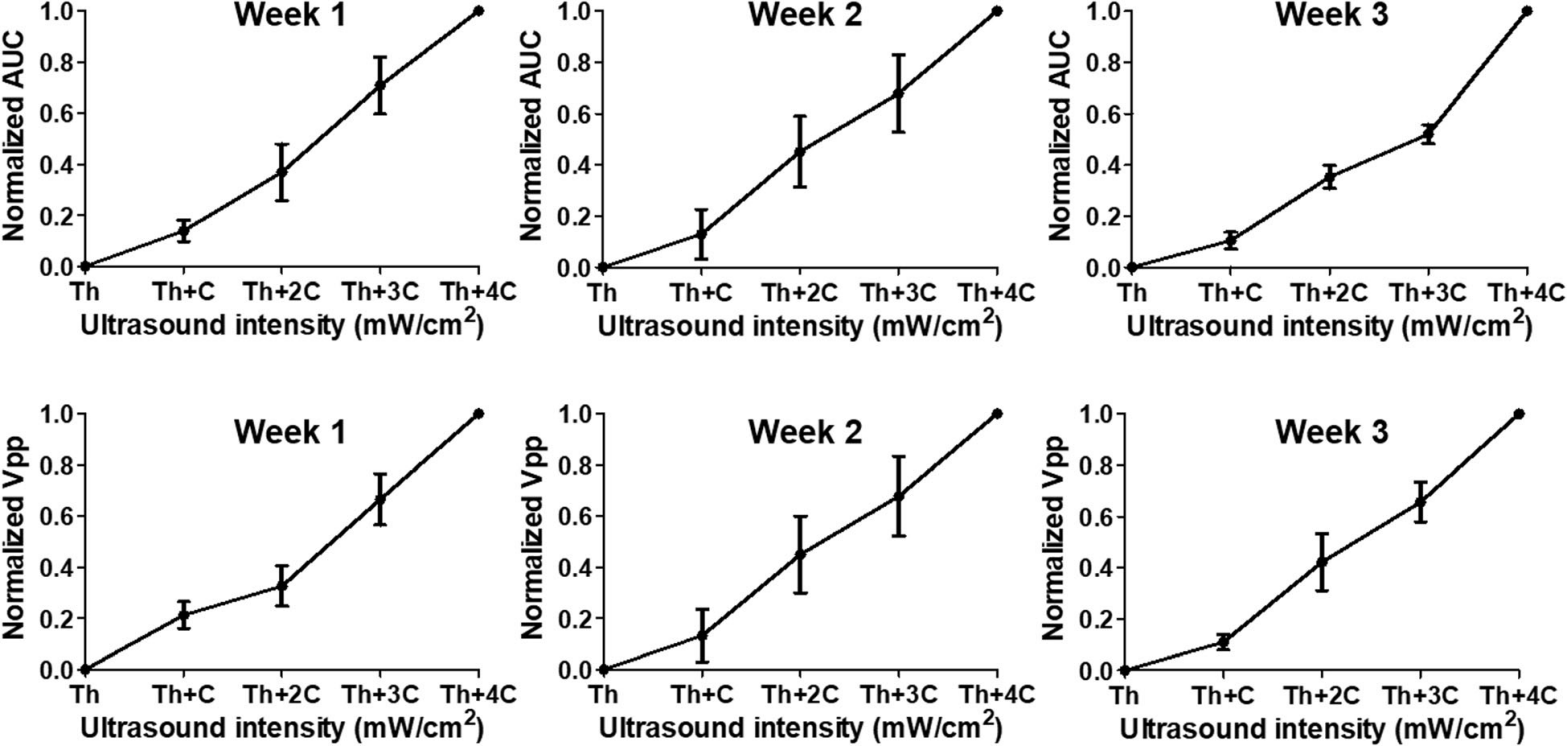
Normalized AUC and V_pp_ of MEPs collected from all healthy rats (*n* = 5) for 3 weeks post-surgery. Recruitment curves of AUC and V_pp_ with increase of acoustic intensity indicate increase in movement strengths via pES current. C equals 0.003 mW/cm^2^ for pES

To further quantify the evoked responses induced by the spinal cord stimulation, AUC and V_pp_ values were normalized and plotted at threshold (Th) and constant (C) increment intensities of 0.1 V (for ES) and 0.003 mW/cm^2^ (for pES) (see Fig. 4). Although the data at week two shows a slightly higher response for 2nd and 3rd increments (Th + C and Th + 2C) with pES, the graphs of ES and pES nearly overlap each other, suggesting similar recruitment of TA muscles for ES and pES. Figure 5 shows overall muscle recruitment with the piezoelectric stimulation (pES) for all the rats (*n* = 5). It indicates that larger muscle recruitment could be observed by increasing ultrasound intensity.

**Fig. 6:**
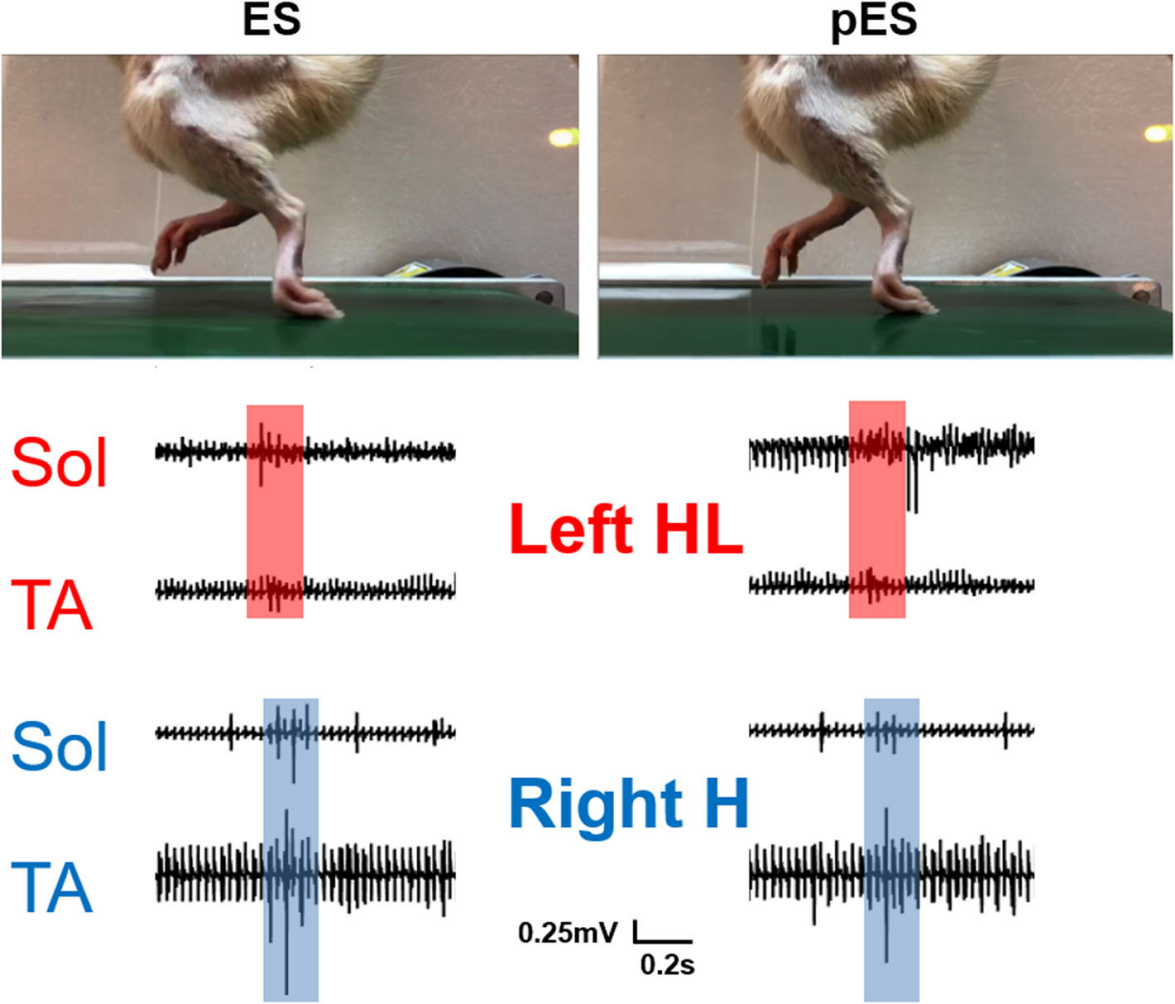
Demonstration of a paralyzed rat for successful movement restoration of hindlimbs on a moving treadmill belt during conventional ES and our pES pulses at 40 Hz. EMG signals were recorded from the soleus and TA muscles for both ES and pES, and are shown within one complete gait cycle

Thoracic cord transection of a rat leads to complete paralysis of the hindquarters as a result of neuronal disconnection to and from the supraspine. The hindlimb locomotion is completely lost due to this injury and the rats cannot feel or move their hindlegs. However, with proper training and lumbosacral stimulation, the rat can regain some involuntary locomotion on a moving treadmill belt (Edgerton et al. 2008; Shah et al. 2016). In the present study, we examined two Sprague Dawley rats with complete spinal cord transection at the T8 level to restore locomotion on a moving treadmill belt with ES or pES. The rats were trained to walk bipedally on a treadmill for 2 weeks before spinal transection. We found that both ES and pES could restore locomotion like movements of the rats’ hindlimbs during either stimulation (Supplementary video 1). EMG signals for an entire gait cycle during locomotion elicited by ES and pES exhibited consistency (see Fig. 6). The performance for ES and pES appeared consistent suggesting the clinical translation of our piezostimulator for movement restoration after paralyzing SCI.

In our experiments, we did not notice any significant displacements of the implanted stimulator while placing the external ultrasound probe. We found that in chronic stage, fat and connective tissue grow around the stimulator and kept it in a fixed positing. However, it is worthwhile to note that it is rather difficult to keep the probe well placed on the skin during the locomotion. In future application, this point needed to be considered while designing an external ultrasound probe for successful clinical translation.

## Conclusions

ES has played an important role in bioelectronic medicine such as on reducing neuropathic pain or restoring functional movements after paralysis. For conventional ES treatment, a bulky battery powered neurostimulator is required to be implanted which poses significant technical and safety concerns for patients. In the present study, we have demonstrated that pES delivered by our develop piezostimulator can achieve the same as ES without requiring a battery. Hence, pES offers a new form of electroceutical with features overtaking conventional ES while keeping the same functionality.

## Supplementary information

**Additional file 1: Supplementary Table 1.** Center frequencies of the piezoelectric ceramics. **Supplementary Figure 1.** Hydrophone (Onda HNP-1000, ONDA Corporation, United States) setup for ultrasound intensity measurements in a water tank.

## Data Availability

Please contact the corresponding authors for data request.

## Abbreviations

WHO: World Health Organization
SCI: Spinal cord injury
FES: Functional electrical stimulation
ES: Electrical stimulation
CPG: Central pattern generator
pES: Piezoelectric stimulation
Sol: Soleus muscle
TA: Tibialis anterior muscle
MEPs: Motor evoked potentials
V_pp_: Peak-to-peak voltage
AUC: Area-under-the-curve
PRF: Pulse repetition frequency
I_SPTA_: The special-peak temporal-average intensity
I_SPPA_: The special-peak pulse-average intensity
MI: Mechanical Index
FDA: Food and Drug Administration
ER: Early response
MR: Middle response
LR: Late response
Th: Threshold
C: Constant
