# Supplementary material for: Ultrasound-driven piezoelectric current activates spinal cord neurocircuits and restores locomotion in rats with spinal cord injury
Source: Bioelectron Med. 2020 Jun 1;6:13. doi: 10.1186/s42234-020-00048-2 (PMC7268413; doi:10.1186/s42234-020-00048-2)
Supplement: Supplementary file 2 — Additional file 1: Supplementary Table 1. Center frequencies of the piezoelectric ceramics. Supplementary Figure 1. Hydrophone (Onda HNP-1000, ONDA Corporation, United States) setup for ultrasound intensity measurements in a water tank. [file 42234_2020_48_MOESM1_ESM.docx]

**Supplementary table 1.** Center frequencies of the piezoelectric ceramics.

| Piezo element | 1 | 2 | 3 | 4 | 5 | 6 | 7 |
| --- | --- | --- | --- | --- | --- | --- | --- |
| Frequency of  peak-value (MHz) | 0.98 | 1.00 | 1.02 | 0.99 | 0.97 | 0.99 | 0.99 |


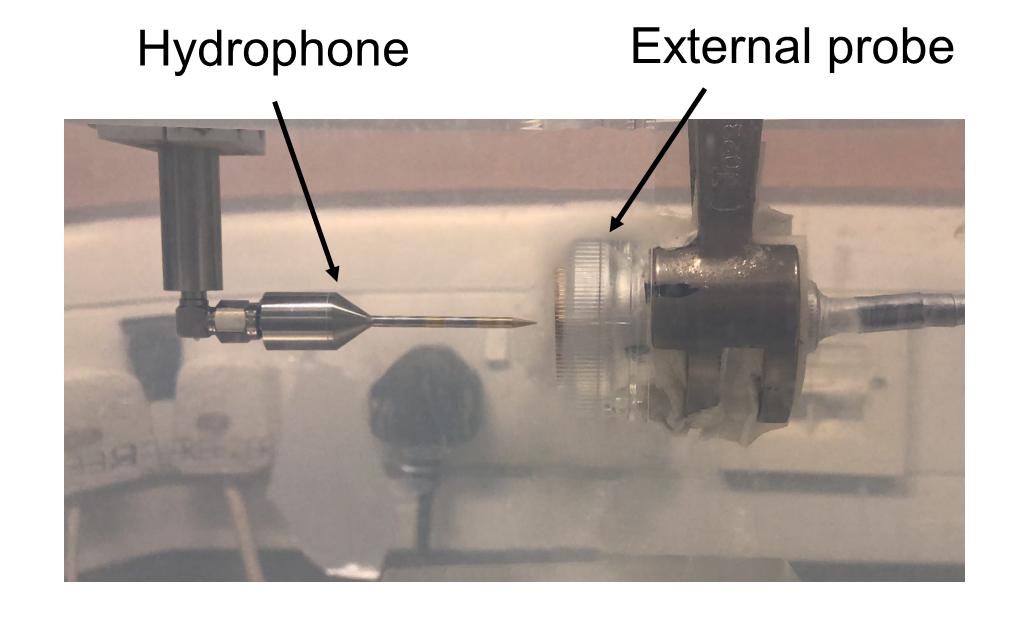


**Supplementary Figure 1.** Hydrophone (Onda HNP-1000, ONDA Corporation, United States) setup for ultrasound intensity measurements in a water tank.
